# Early fixation versus conservative therapy of multiple, simple rib fractures (FixCon): protocol for a multicenter randomized controlled trial

**DOI:** 10.1186/s13017-019-0258-x

**Published:** 2019-07-30

**Authors:** Mathieu M. E. Wijffels, Jonne T. H. Prins, Suzanne Polinder, Taco J. Blokhuis, Erik R. De Loos, Roeland H. Den Boer, Elvira R. Flikweert, Albert F. Pull ter Gunne, Akkie N. Ringburg, W. Richard Spanjersberg, Pieter J. Van Huijstee, Gust Van Montfort, Jefrey Vermeulen, Dagmar I. Vos, Michael H. J. Verhofstad, Esther M. M. Van Lieshout

**Affiliations:** 1 Trauma Research Unit Department of Surgery, Erasmus MC, University Medical Center Rotterdam, P.O. Box 2040, 3000 CA Rotterdam, The Netherlands; 2 Department of Public Health, Erasmus MC, University Medical Center Rotterdam, P.O. Box 2040, 3000 CA Rotterdam, The Netherlands; 3 Department of Surgery, Maastricht University Medical Center, P.O. Box 5800, 6202 AZ Maastricht, The Netherlands; 4 Department of Surgery, Zuyderland Medisch Centrum, P.O. Box 5500, 6130 MB Sittard-Geleen, The Netherlands; 5 Department of Surgery, Spaarne Gasthuis, P.O. Box 417, 2000 AK Haarlem, The Netherlands; 6 Department of Surgery, Deventer Ziekenhuis, P.O. Box 5001, 7400 GC Deventer, The Netherlands; 7 Department of Surgery, Rijnstate, P.O. Box 9555, 6800 TA Arnhem, The Netherlands; 8 Department of Surgery, Ikazia Ziekenhuis, P.O. Box 5009, 3008 AA Rotterdam, The Netherlands; 9 Department of Surgery, Isala, P.O. Box 10400, 8000 GK Zwolle, The Netherlands; 10 Department of Surgery, Haga Ziekenhuis, P.O. Box 40551, 2504 LN The Hague, The Netherlands; 11 Department of Surgery, Catharina Ziekenhuis, P.O. Box 1350, 5602 ZA Eindhoven, The Netherlands; 12 Department of Surgery, Maasstad Ziekenhuis, P.O. Box 9100, 3007 AC Rotterdam, The Netherlands; 13 Department of Surgery, Amphia Ziekenhuis, P.O. Box 90158, 4800 RK Breda, The Netherlands

**Keywords:** Rib fractures, Non-flail rib fractures, Operative fixation, Nonoperative treatment, Pneumonia, Cost-effectiveness, Quality of life, RCT, Randomized controlled trial

## Abstract

**Background:**

Multiple rib fractures are common injuries in both the young and elderly. Rib fractures account for 10% of all trauma admissions and are seen in up to 39% of patients after thoracic trauma. With morbidity and mortality rates increasing with the number of rib fractures as well as poor quality of life at long-term follow-up, multiple rib fractures pose a serious health hazard. Operative fixation of flail chest is beneficial over nonoperative treatment regarding, among others, pneumonia and both intensive care unit (ICU) and hospital length of stay. With no high-quality evidence on the effects of multiple simple rib fracture treatment, the optimal treatment modality remains unknown. This study sets out to investigate outcome of operative fixation versus nonoperative treatment of multiple simple rib fractures.

**Methods:**

The proposed study is a multicenter randomized controlled trial. Patients will be eligible if they have three or more multiple simple rib fractures of which at least one is dislocated over one shaft width or with unbearable pain (visual analog scale (VAS) or numeric rating scale (NRS) > 6). Patients in the intervention group will be treated with open reduction and internal fixation. Pre- and postoperative care equals treatment in the control group. The control group will receive nonoperative treatment, consisting of pain management, bronchodilator inhalers, oxygen support or mechanical ventilation if needed, and pulmonary physical therapy. The primary outcome measure will be occurrence of pneumonia within 30 days after trauma. Secondary outcome measures are the need and duration of mechanical ventilation, thoracic pain and analgesics use, (recovery of) pulmonary function, hospital and ICU length of stay, thoracic injury-related and surgery-related complications and mortality, secondary interventions, quality of life, and cost-effectiveness comprising health care consumption and productivity loss. Follow-up visits will be standardized and daily during hospital admission, at 14 days and 1, 3, 6, and 12 months.

**Discussion:**

With favorable results in flail chest patients, operative treatment may also be beneficial in patients with multiple simple rib fractures. The FixCon trial will be the first study to compare clinical, functional, and economic outcome between operative fixation and nonoperative treatment for multiple simple rib fractures.

**Trial registration:**

www.trialregister.nl, NTR7248. Registered May 31, 2018.

## Background

Rib fractures are common injuries in both trauma and non-trauma centers, occurring in up to 10–39% of patients with blunt chest trauma and accounting for 10% of all trauma admissions [1–4]. With an estimated 25% of all traumatic deaths, chest trauma ranks second after head injury [3, 4]. Rib fractures are caused by high-energy trauma (HET) in the younger patients, often with concomitant injuries, and in the elderly as a result of low energy trauma (LET) [5–7]. Sustaining multiple rib fractures can result in a flail chest, defined as fracture of three or more consecutive ribs in two or more places, creating a flail segment [8, 9]. Patients may also have multiple simple rib fractures or a combination of both.

While open surgical fixation of rib fractures dates back to the 1940s, multiple rib fractures are routinely treated nonoperatively [10]. Nonoperative treatment includes pain management, oxygen support or mechanical ventilation, bronchodilator inhalers, and pulmonary physical therapy. Despite this treatment strategy, mortality and complications such as pulmonary contusion, hemopneumothorax, and pneumonia are seen in up to 34% and in 35–77% of patients, respectively [1, 2, 5, 6, 11–15]. Various studies have identified risk factors that increase mortality such as age and number of rib fractures [3, 6, 7, 11, 16–18].

Furthermore, at 2 years post-injury, up to 29% of patients have not yet returned full time to their pre-injury job and 64% of patients with isolated multiple rib fractures still experience chest wall pain [19, 20]. With incapacitating pain often accompanying traumatic rib fractures, epidural analgesics are suggested as the optimal analgesic for patients with multiple rib fractures. Two meta-analyses have shown that epidural use results in significant less pain but has no benefit regarding the length of both intensive care unit (ICU) and hospital stay, mortality, and complication rate, indicating the necessity of an optimized analgesic modality for rib fracture patients [21, 22].

Rib fractures may show the same pattern as a restrictive pulmonary disease, resulting in loss of total lung capacity which precipitates inadequate oxygenation and ventilation. Patients with rib fractures and reduced pulmonary function are more susceptible to pulmonary complications and longer length of hospital stay [23–25]. With contradicting studies on the difference in spirometry between operatively and nonoperatively treated patients with rib fractures, additional research is needed [12–14, 26–29]. While surgical treatment of flail chest patients appears to be cost-effective over nonoperative treatment, but for multiple simple rib fractures, the most cost-effective treatment modality is still unknown [30, 31].

Over the last decade, there has been an increasing number of studies suggesting superior results of open reduction and fixation (ORIF) for the stabilization of multiple rib fractures due to profitable results in traumatic flail chests compared with nonoperative management [31–34]. Several studies with flail and non-flail chest patients combined have shown promising effects of ORIF with less pneumonia, less hemo- and pneumothorax, shorter need for mechanical ventilation, lower mortality, shorter length of hospital and ICU stay, and quicker return to normal activity [15, 28, 35–38].

As only two studies, both retrospective cohort studies with small sample sizes and short follow-up, have singularly focused on operative versus nonoperative management of multiple simple rib fractures, definitive proof for the optimal treatment of multiple simple rib fractures is not achieved yet [9, 14, 39–41].

Therefore, the aim of this multicenter randomized controlled trial is to investigate the effect of ORIF versus nonoperative treatment in patients who sustained multiple simple fractured ribs.

## Methods/design

### Objective

The primary aim of this trial is to investigate the effect of ORIF versus nonoperative treatment on the occurrence of pneumonia within 30 days after trauma in adult patients who sustained multiple simple fractured ribs. The secondary aims are to investigate the effect of treatment on the need for and duration of mechanical ventilation, level of thoracic pain and analgesics use, (recovery of) pulmonary function, hospital and ICU length of stay, thoracic injury-related and surgery-related complications and mortality, secondary interventions, quality of life, and total costs (in-hospital and socio-economic) of treatment, health care consumption, and work absence. At the end, a cost-effectiveness analysis will be done.

### Trial design and setting

The FixCon trial is a multicenter randomized controlled trial, with a parallel group design. The following 12 hospitals in The Netherlands will participate: Amphia Ziekenhuis (Breda), Catharina Ziekenhuis (Eindhoven), Deventer Ziekenhuis (Deventer), Erasmus MC (Rotterdam), Haga Ziekenhuis (The Hague), Ikazia Ziekenhuis (Rotterdam), Isala (Zwolle), Maasstad Ziekenhuis (Rotterdam), Maastricht UMC+ (Maastricht), Rijnstate (Arnhem), Spaarne Gasthuis (Haarlem), and Zuyderland Medisch Centrum (Heerlen).

## Inclusion and exclusion criteria

The study population will consist of adults with three or more simple rib fractures after blunt force trauma. The fracture pattern will be diagnosed and delineated with a CT scan of the thorax, at least 64-slice and preferable including 3D reconstruction.

In order to be eligible to participate in this study, a patient must meet all of the following inclusion criteria:

1. Age 18 years or older
2. For any of the ribs number 4 to 10, three simple fracture ribs with either A) at least one fracture dislocated over one shaft-width; or B) unbearable pain (VAS or Numeric Rating Scale (NRS) >6 points)
3. Blunt force trauma
4. Hospital presentation within 72 h after trauma
5. Provision of informed consent by patient or proxy

A patient who meets any of the following criteria will be excluded from participation:

1. Neurotraumatic changes leading to mechanical ventilation (GCS ≤ 8 at 48 h post-injury. If unable to assess full GCS due to intubation or other causes, GCS motor ≤ 4 at 48 h post-injury)
2. Rib fractures due to cardiopulmonary resuscitation
3. Surgical rib fixation not possible due to additional traumatic injuries (hemodynamically or pulmonary unstable, for example, based on parenchymal lung trauma) or the patient is unfit for surgery, to be decided by an ICU doctor, trauma surgeon, or anesthesiologist
4. Flail chest, based on radiological or clinical findings
5. Decreased sensory or motor function due to (previous) cervical or thoracic spine failure
6. Previous rib fractures or pulmonary problems, requiring continuous oxygen use at home pre-trauma
7. Congenital thoracic deformity (pectus excavatum, pectus carinatum, severe scoliosis, or kyphosis)
8. Inhalation trauma or severe burns close to or inside the mouth or neck
9. Surgical fixation of the ribs not feasible within 7 days after trauma
10. Patient unwilling or unable to comply with the intervention or follow-up visit schedule
11. Insufficient comprehension of the Dutch language to understand the rehabilitation program and other treatment information in the judgement of the attending physician
12. Participation in another surgical intervention or drug study that might influence any of the outcome parameters

### Recruitment and randomization

Eligible persons presenting to the emergency department (ED) or referred from another hospital, with multiple, simple rib fractures will be informed about the trial at the ED or at the surgical ward after admission. After explanation of the study, eligible patients will receive written information and a consent form from the attending physician, the clinical investigator, or a research assistant. Patients meeting all eligibility criteria will be recruited within 1 day after hospital admission. As surgical rib fixation appears to be most beneficial when performed within 72 h after trauma, patients are stimulated to decide within this period. However, informed consent can be given by the patient as long as rib fixation can be carried out within 1 week after trauma. Should patients not be able to sign informed consent themselves, a legal representative will receive oral and written information about the study, in the hospital, by the attending physician, the clinical investigator, or a research assistant, and will be asked to consent with participation of the patient.

After signing informed consent by patient or proxy, participants are allocated to one of the two study arms (surgical stabilization or nonoperative treatment) using a web-based randomization program that will be available 24 h a day. Allocation will be at random and concealed, in a 1:1 ratio, and will be stratified by site. Variable block sizes will be used; in each block, both treatments will be represented equally. As the intervention cannot be blinded, it will in no case be necessary to break the randomization code.

As with many surgical trials, patients and surgeons cannot be blinded for the intervention. In order to reduce bias as much as possible, a research physician or research assistant will perform the follow-up measurements using a standardized protocol. Also, the treating surgeon or ICU doctor will identify the primary outcome (i.e., pneumonia) based on the definition as mentioned under outcome measures.

Participation is on a voluntary basis and participants are allowed to withdraw from the study at any time without specifying why. The general practitioner will be informed about the patients’ participation.

## Nonoperative allocation

Nonoperative treatment will consist of optimal pain treatment, supportive oxygen or ventilation if needed, early mobilization, Salbutamol/Atrovent spray, and physical therapy for optimizing ventilation. Without definitive proof for the best protocol, each participating center is allowed to use its local protocol for interpleural drainage use, mechanical ventilation, and pain control. Although this may introduce some heterogeneity across hospitals, it benefits extrapolation of the results. Critical elements of the nonoperative treatment will be recorded.

## Operative allocation

Preoperative treatment is the same as in the nonoperative treatment group. ORIF should be preferably carried out within 72 h after trauma, but fixation within 1 week will not lead to exclusion. The surgical fixation will be conducted by a senior fracture management surgeon who has participated in at least five rib fracture fixation procedures. A surgeon in training with limited experience in rib fixation is allowed to work under supervision of an experienced surgeon.

Patients allocated to the surgical group will undergo ORIF using plates and/or splints. The decision on what rib fixation system to use is to the discretion of the treating surgeon, provided that the fixation system is CE-mark approved for rib fixation. Each system will be used according to the supplier’s protocol. The patient will receive an intravenous single prophylactic dose of a third-generation cephalosporin preoperatively. The incision will be planned, based preferably on a preoperative 3D reconstruction of the thoracic cage. The positioning of the patient and number of ribs fixated will be left to the preference of the operating surgeon. A minimally invasive technique will not lead to exclusion of the study. The ribs will be visualized using a muscle-sparing approach. After removing interpositioning tissue, fracture reduction will be carried out and the rib fixation device will be positioned and fixated. The use of interpleural space rinsing with warmed NaCl 0.9% or thoracoscopic visualization during rib fixation will be left to the judgement of the surgeon. If indicated, an interpleural drain is percutaneously placed in dorsocaudal direction, apart from the surgical wound. The wound is closed, using a wound drain if needed.

After surgery, the patient will be admitted to the ward or ICU depending on his/her clinical state. Participating hospitals are allowed to use their local protocol for interpleural and wound drainage. Postoperative physical therapy and supportive treatment may be prolonged if needed. Postoperative care and preoperative treatment are the same as for nonoperative management. Critical elements of the operative treatment will be recorded.

### Outcome measures

#### Primary outcome measure

**Fig. 1:**
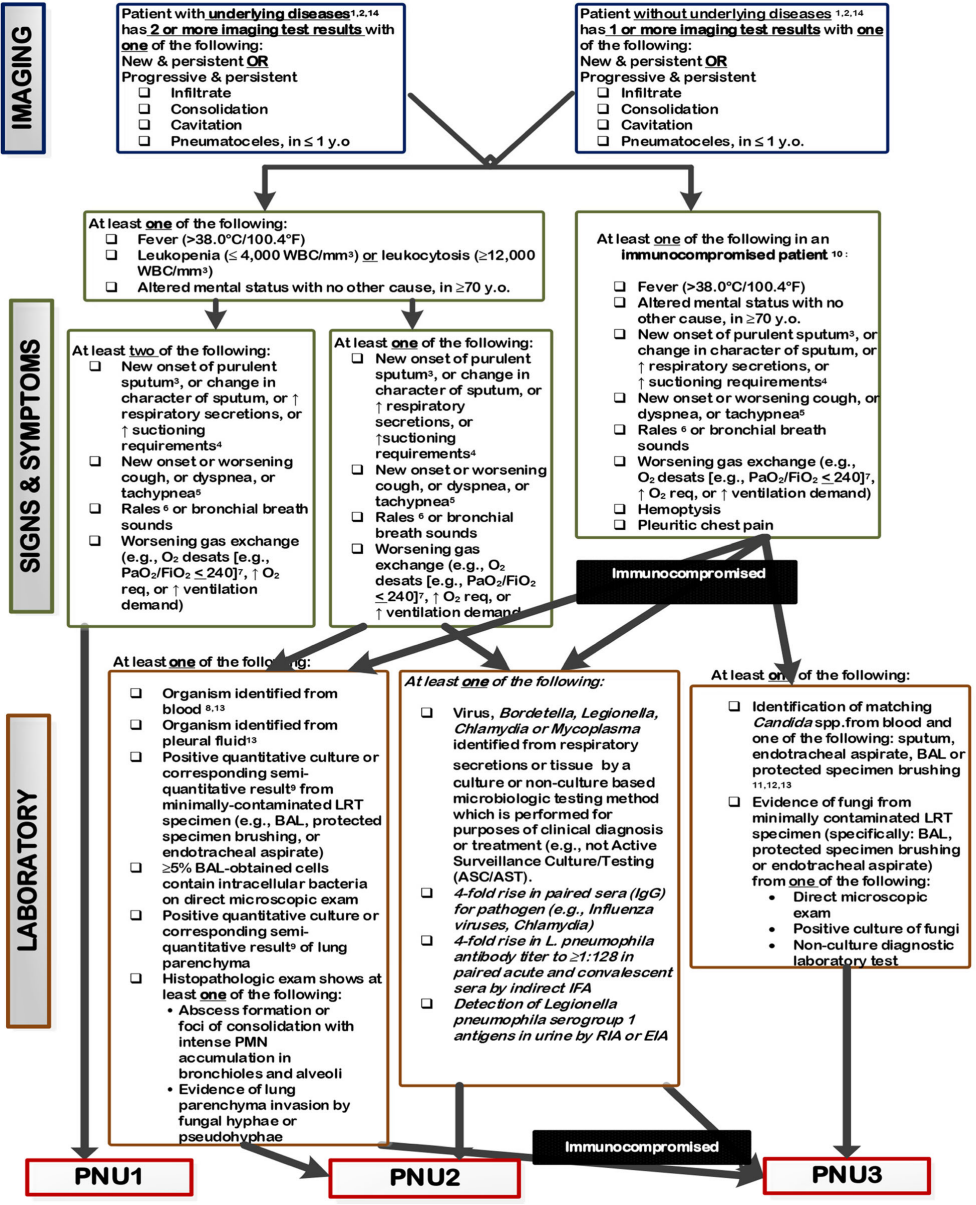
Pneumonia flow diagram, as designed by the CDC [42]. For further details of the flow chart, see the website of the CDC [42]

The primary outcome measure is pneumonia within 30 days after trauma. In order to define pneumonia, the flowchart of the Centers of Disease Control and Prevention, based on imaging and clinical and laboratory criteria, will be followed (Fig. 1) [42]. Temperature (*T*) will be measured daily during admission. If *T* > 38.0 °C intra-auricular (or *T* > 39 °C rectal), bladder, central, or a sputum culture will be done. Also, the wound will be checked (if applicable) and a radiograph of the thorax will be made. If patients are suffering from fever at home, they will be advised to visit the outpatient clinic or emergency department. The temperature will be measured on arrival at the outpatient clinic or emergency department, and the same additional examinations will be performed. A monitor will independently review the patient’s medical files in order to ensure that the pneumonia was actually present.

#### Secondary outcome measures

The secondary outcome measures are:

1. Need and duration of mechanical ventilation in days. The number of days of invasive mechanical ventilation (by endotracheal tube or tracheostomy) from intubation until successful weaning will be determined. The need for mechanical ventilation will be evaluated based on arterial blood gas analysis and clinical performance of the patient. The duration of mechanical ventilation will be calculated from the dates of intubation and extubation. Re-intubation within 30 days will be recorded as well.
2. Level of thoracic pain (NRS) and analgesics use. Thoracic pain will be determined using an 11-point Numeric Rating Scale (NRS) in which 0 implicates no pain and 10 the worst possible pain. Five thoracic pain levels will be analyzed: pain in rest, at night, during daily activities (e.g., work or hobbies), during maximal inspiration, and during self-care. Analgesics use during admission will be extracted from the medical files. After discharge, analgesics use will be asked for during the follow-up visits. Daily narcotic requirement will be calculated using an equivalence scale for 30 mg/day oral morphine.
3. (Recovery of) pulmonary function. The parameters tidal volume (TV), forced vital capacity (FVC), inspiratory capacity (IC_lung_), and forced expiratory volume at 1 second (FEV_1_) will be determined using spirometry. Spirometry will be done by a member of the research team. During the spirometry, the patient has to inhale actively and exhale with maximum force possible. The mean of three tests will be calculated.
4. Hospital length of stay expressed in days. This will be calculated as the time between admission and discharge from the hospital. Re-admission within 30 days will be added. Re-admission for thoracic reasons after 30 days and up to 12 months will be counted separately.
5. ICU length of stay expressed in days. This will be calculated similarly to the hospital length of stay.
6. Thoracic injury-related complications and mortality. The occurrence of thoracic injury-related complications will be recorded from the medical charts during clinical admission and each follow-up visit. Complications will be categorized for level of severity and treatment necessity according to the Clavien-Dindo classification [43]. Complications will include empyema (as diagnosed on CT scan, in presence of fever or positive cultures in the drained fluid), (retained) hydrothorax (a heterogeneous fluid collection with Hounsfield unit readings of 35–70 and evidence of pleural thickening) [44], nonunion (diagnosed on CT scan or operatively, at least 6 months after trauma) [45], and other (all other complications as judged by the treating physician). If mortality is caused by the thoracic injury or complication of thoracic injury, it will be counted in rates of mortality. Death caused by other reasons will be noted but excluded in this calculation.
7. Surgery-related complications. The ORIF group can also develop hardware-related complications or failure. Hardware-related complication is superficial and deep wound infection which is defined as redness, tenderness, and warmth surrounding and in direct contact with the postoperative wound. Superficial infection leads to oral or IV antibiotics, and deep infection leads to surgical activity such as stitch removal of exploration of the wound. Hardware failure is defined as loosening of the plate, secondary dislocation of fixation material, malposition of hardware, and broken plates or splints.
8. Secondary interventions to resolve complications. Secondary interventions within 12 months after trauma to relieve pain, treat infection, or other rib fracture-related problems will include the following: antibiotic therapy (both oral and intravenous), additional surgical interventions (e.g., surgical stabilization of nonunion, evacuation of hematoma, evacuation of empyema, removal of failed hardware, symptomatic hardware removal, and treatment of infection), and additional percutaneous interventions (e.g., for persistent bleeding intercostal artery, intraparenchymal bleeding, drainage of infection, and drainage of pleural fluid).
9. Health-related quality of life measured using the Short Form-12 (SF-12) and EuroQoL-5D (EQ-5D) questionnaires. The SF-12 analyzes global health status, functional scale, and symptom scale. The score will be calculated based on eight domains and summarized into a Physical Component Summary (PCS) and Mental Component Summary (MCS). Data will be reported as utility score, ranging from 0 to 1 with a higher value indicating better quality of life. As a reference, the US population of 1998 will be used [46]. The EQ-5D is the most commonly used quality of life instrument for (rib) fracture patients [47, 48]. The EQ-5D is recommended for assessment of quality of life in trauma patients, especially for economic evaluation [49, 50]. The EQ-5D-5 L descriptive system consists of five dimensions of health (mobility, self-care, usual activities, pain/discomfort, and anxiety/depression), each with five possible answers. The patients’ health states will be converted into a utility score using the Dutch tariff [51]. Utility scores range from 0 to 1 with lower scores indicating poorer quality of life.
10. Cost-effectiveness and health care consumption. Economic evaluations will be done from a societal perspective. The validated Medical Consumption Questionnaire (iMCQ) and Production Consumption Questionnaire (iPCQ) will be used. iMCQ details medical specialist care, physical therapy, hospitalization, nursing home, home care, and other costs directly associated with diagnosis, treatment, and rehabilitation. iPCQ comprises work resumption and production losses. Health care costs and lost productivity until 1 year after trauma will be measured in accordance with economic guidelines [52].

### Other data collected

In addition to the outcome measures, the following data will be collected in order to assess similarity between the treatment groups:

Intrinsic variables (baseline characteristics): age, gender, body mass index (BMI), American Society of Anesthesiologists (ASA) grade, tobacco consumption, comorbidities, and medication use.

Injury-related variables: injury mechanism, pleura drain placed, number and location of rib fractures, affected side, presence of sternum fracture, additional injuries represented by the Abbreviated Injury Score (AIS) [53], and Injury Severity Score (ISS).

Intervention-related variables: surgical approach, number of plates and splints used and for which ribs, surgical delay, primary and secondary surgeon (resident or staff surgeon), wound drain, intra-operatively placed interpleural drain including duration of drainage, and duration of surgery.

### Study procedures

Patients will be followed until 12 months after trauma. Clinical evaluation will occur daily during hospital admission. After discharge, outpatient clinic evaluation will occur at 2 weeks (window 7–21 days), 1 month (window 21–39 days), 3 months (window 11–15 weeks), 6 months (window 24–28 weeks), and 12 months (window 12–14 months). These visits are standard of care for the targeted patient group. A schedule of events is shown in Table 1. Baseline data and perioperative data will be collected from the patients’ medical files as soon as possible, but no later than the first outpatient department visit. At the 12-month follow-up contact, the surgeon or research assistant will document any secondary intervention that may be planned for the patient.

**Table 1:** Schedule of events (duration after trauma)

Radiographs and event forms	Screening	Enrolment	Pre-surgery	Surgery	Post-surgery (until day 7*)	2 weeks (7–21 days)	30 days (21–39 days)	3 months (11–15 weeks)	6 months (24–28 weeks)	12 months^$^ (12–14 months)
CT-scan	X								X	
Screening	X									
Informed consent		X								
Randomization		X								
Baseline data			X	X						
Intervention/surgical report form				X	X					
Outpatient clinic FU						X	X	X	X	X
Spirometry							X	X	X	X
Analgesic use			Daily, afternoon		Daily, afternoon	X	X	X	X	X
Pain (NRS-rest and inspiration)			Daily, afternoon		Daily, afternoon	X**	X	X	X	X
Pain (NRS-night, daily, and care)						X**	X	X	X	X
QoL (EQ-5D and SF-12)						X**	X	X	X	X
Complications			X	X	X	X	X	X	X	X
(Secondary) interventions			X		X	X	X	X	X	X
iPCQ and iMCQ questionnaire						X***	X	X	X	X
Early withdrawal						****	****	****	****	****

*Post-surgery

^$^May be planned at the patients’ residency

**Asking for current and pre-trauma status

***Asking for pre-trauma situation

****Only if applicable.

After 6 months, a thoracic CT scan is repeated. Pulmonary function will be tested during the outpatient clinic visits at 1, 3, 6, and 12 months. At each follow-up visit, the coordinating researcher or research assistant will ascertain patient status (i.e., adverse events/complications or secondary interventions) and will verify information within the medical records. At each visit, patients will be asked to complete questionnaires relating to their pain (NRS), analgesics use, quality of life (QoL) (SF-12 and EQ-5D), and health care use (iPCQ and iMCQ).

### Sample size calculation

Calculation of the required sample size for the primary analysis is based on data from a Cochrane review and a large retrospective analysis [7, 32]. These studies suggest a pneumonia rate of 35% in nonoperatively treated patients and 15% in operatively treated patients with multiple rib fractures. This difference is considered clinically relevant. A two-sided test with an α level of 0.05 and a β level of 0.2 requires 72 patients in each group. In order to account for 25% loss of patients to follow-up and mortality, a sample size of 90 patients per group is needed. In total, 180 patients will be included and randomized.

### Statistical analyses

Data will be analyzed using the Statistical Package for the Social Sciences (SPSS), version 24.0 or higher (SPSS, Chicago, IL, USA), and reported following the Consolidated Standards of Reporting Trials (CONSORT) guidelines. Normality of continuous data will be tested with the Shapiro-Wilk test. Homogeneity of variances will be tested using Levene’s test. The analysis will be performed on an intention to treat basis. A two-sided *p* value < 0.05 will be taken as threshold of statistical significance in all statistical tests. Procedures will be implemented to reduce missing data. In previous studies of the principal investigator’s department, these procedures led to < 5% missing data [54]. If necessary, missing values will be replaced using multiple imputation following the predictive mean matching method, using ten imputations.

Descriptive analysis will be performed in order to report the outcome measures for both treatment arms. For categorical data, numbers and frequencies will be reported. For continuous data, the mean and SD (parametric data) or the mean and percentiles (non-parametric data) will be reported.

Next, univariate analysis will be performed in order to test for statistical significance of differences between the primary and secondary outcome measures across the two groups. A chi-squared analysis or Fisher’s exact test will be used for statistical testing of categorical data (e.g., the primary outcome, pneumonia). Continuous data (i.e., hospital length of stay) will be tested using Student’s *t* test (parametric data; with equal variance or unequal variance whichever applies according to Levene’s test) or Mann-Whitney *U* test (non-parametric data).

Multivariable analysis will be done as secondary analysis. A logistic regression model will be developed, with pneumonia as dependent variable and treatment as covariate. Nonoperative treatment will serve as reference category. Baseline and injury-related variables that may potentially confound the association between treatment and outcome will be included in this model as covariate. These will be selected from literature and from data of this study (see Other data collected). Known potential confounders according to literature data are the number of rib fractures and age. Other potential confounders collected as part of this study are gender, ASA, COPD, osteoporosis, and additional injuries (ISS ≥ 16 versus ISS < 16, and presence versus absence of severe injuries (AIS ≥ 3) for any body region). Variables that produce a *p* value < 0.2 in the univariate analysis will be included in the regression model. Odds ratios will be reported with their 95% confidence interval and *p* value.

Continuous outcomes repeatedly measured over time will be compared between treatment groups using linear mixed-effects regression models (with fixed effects for treatment and other covariates like gender and age, if applicable). The interaction between treatment and time will be included to test for differences between groups over time. For each follow-up moment, the estimated marginal mean will be computed per treatment group and compared post hoc with a Bonferroni test in order to correct for multiple testing.

Other continuous and binomial variables will be tested with multivariable linear and binary logistic regression models, respectively. The outcome measure will be entered as dependent variable and treatment as covariate. Nonoperative treatment will serve as the reference group. Baseline and injury-related variables that may potentially confound the association between treatment and outcome will be included in the models as covariate. Coefficients will be reported with their 95% confidence interval and *p* value.

Economic evaluation will be done from a societal perspective with iMCQ and iPCQ questionnaires. Health care costs and lost productivity until 1 year after trauma will be measured. Cost prices of the standardized referral strategy will be determined by a bottom-up micro-costing method. The incremental cost-effectiveness ratio of ORIF versus nonoperative treatment will be expressed as costs per pneumonia prevented, with confidence ellipses and acceptability curves. A cost-utility analysis, with QALY (based on the EQ-5D summary score) as outcome measure, will also be done.

## Ethical concerns

The study will be conducted according to the principles of the Declaration of Helsinki (64th World Medical Association General Assembly, Fortaleza, Brazil, October 2013) and in accordance with the Medical Research Involving Human Subjects Act (WMO). This study has been approved by the Medical Research Ethics Committee (MREC), in Dutch: Medisch Ethische Toetsings Commissie (METC). The MREC Erasmus MC has given dispensation from the statutory obligation to provide insurance for subjects participating in medical research (article 7 of the WMO and Medical Research (Human Subjects) Compulsory Insurance Decree of 23 June 2003) as participation involves no risks.

Participants can leave the study at any time for any reason if they wish to do so without having to give a reason. No replacement will take place. Anticipated loss to follow-up is included in the sample size calculation. Reasons for non-participation will be documented.

## Data management and monitoring

Data will be encoded and stored in a password-protected database (Data Management, The Research Manager, Deventer, The Netherlands) with restricted access to the researchers only. Data will be entered once. Quality of the entered data will be monitored by checking entry for a random sample of patients prior to database locking.

## Trial status

The trial is registered at the Netherlands Trial Register (NTR) (NTR7248), registration date May 31, 2018. Inclusion of patients has started January 1, 2019, and the planned recruitment period will be 3 years. With a follow-up of 1 year, data presentation is expected in the beginning of 2022.

## Discussion

The FixCon trial studies outcome after operative versus nonoperative treatment of multiple simple rib fractures. With high rates of morbidity and low quality of life at long-term follow-up, multiple simple rib fractures cause a serious health hazard. With favorable results in flail chest patients, operative treatment might also result in better clinical and functional recovery of patients with multiple simple rib fractures. Improved outcome could translate into less pulmonary complications, shorter hospital stay, less pain, improved quality of life, and quicker return to normal activities or work compared with nonoperative treatment. Operative treatment, while initially yielding higher economic costs, could then result in less financial needs, due to less health care use and less productivity loss. As a result, primarily performing surgery could be both improving patient outcome and being the most cost-effective treatment modality.

To the best of our knowledge, this is the first multicenter randomized controlled trial to evaluate outcome from patient, medical, and economic perspectives in patients suffering from multiple simple rib fractures. Twelve hospitals in the Netherlands will participate in this trial.

## Data Availability

Not applicable as no data have yet been analyzed.

## Abbreviations

AIS: Abbreviated Injury Score
ASA: American Society of Anesthesiologists
BMI: Body mass index
CT: Computed tomography
ED: Emergency department
EQ-5D-5 L: EuroQoL-5D 5 levels
FEV_1_: Forced expiratory volume at 1 second
FVC: Forced vital capacity
GCS: Glasgow Coma Scale
HET: High-energy trauma
IC_lung_: Inspiratory capacity of the lung
ICU: Intensive care unit
iMCQ: International Medical Consumption Questionnaire
iPCQ: International Production Consumption Questionnaire
ISS: Injury Severity Score
LET: Low-energy trauma
MCS: Mental Component Summary
MREC: Medical Research Ethics Committee (in Dutch: Medisch Ethische Toetsings Commissie (METC))
NRS: Numeric Rating Scale
NTR: Netherlands Trial Registry (in Dutch: Netherlands Trial Register)
ORIF: Open reduction and internal fixation
PCS: Physical Component Summary
QALY: Quality-adjusted life year
QoL: Quality of life
RCT: Randomized controlled trial
SD: Standard deviation
SF-12: Short form-12
SPSS: Statistical Package for the Social Sciences
T: Temperature
TV: Tidal volume
US: United States of America
VAS: Visual analog scale
WMO: (in Dutch) Wet medisch-wetenschappelijk onderzoek met mensen (Medical Research Involving Human Subjects Act)
